# Supplementary material for: Manganese co-limitation of phytoplankton growth and major nutrient drawdown in the Southern Ocean
Source: Nat Commun. 2021 Feb 9;12:884. doi: 10.1038/s41467-021-21122-6 (PMC7873070; doi:10.1038/s41467-021-21122-6)
Supplement: Supplementary file 1 — Supplementary Information [file 41467_2021_21122_MOESM1_ESM.pdf]

Supplementary information for: Manganese co-limitation of phytoplankton growth and major nutrient drawdown in the Southern Ocean

Thomas J. Browning[1]*, Eric P. Achterberg[1], Anja Engel[1], Edward Mawji[2]

[1]Marine Biogeochemistry Division, GEOMAR Helmholtz Centre for Ocean Research, Kiel 24148, Germany.

[2]National Oceanography Centre Southampton, Southampton SO14 3ZH, UK.

*Correspondence to: tbrowning@geomar.de

Supplementary Tables

Supplementary Table 1. Experiment starting conditions.

Exp.	Lon. (°E)	Lat. (°N)	Duration (Days)	SST (°C)	$E_{in\ situ}/E_{exp}$†	NO_3^- (μM)	PO_4^{3-} (μM)	$Si(OH)_4$ (μM)	DFe (nM)	DMn (nM)	Mn*(‡) (nM)	Chl.-a (mg m^{-3})
1	-58.01	-54.71	3.1	5.2	1.01	21.07	1.44	6.79	0.35	0.29	0.16	0.82
2	-57.65	-55.44	2.9	5.4	0.57	20.53	1.39	6.39	0.16	0.37	0.31	1.59
3	-57.96	-55.60	1.8	5.1	1.03	21.34	1.45	6.71	0.39	0.36	0.21	0.75
4	-57.79	-55.76	2.9	5.2	0.72	21.49	1.49	7.57	0.25	0.33	0.24	0.91
5	-57.38	-56.55	3.2	2.2	0.66	27.41	1.75	18.85	0.44	0.15	–0.02	0.65
6	-57.23	-56.81	3.2	1.6	0.46	27.38	1.77	20.05	0.39	0.14	–0.01	0.59
7	-56.42	-58.08	5.1	-0.4	1.01	27.31	1.79	36.12	0.34	0.17	0.04	1.21
8	-56.09	-58.66	3.7	-0.2	0.52	26.32	1.57	41.81	0.52	0.38	0.19	2.18
9	-55.47	-59.64	3.1	-0.1	0.97	26.68	1.59	46.40	0.42	0.34	0.18	2.97
10	-54.64	-60.97	3.9	-0.6	1.99	29.83	2.08	78.52	0.78	2.35	2.06	1.26

†Ratio of estimated in situ mean mixed layer irradiance to mean experimental irradiance
‡Mn*=DMn – DFe/$R_{Fe:Mn}$, where $R_{Fe:Mn}$ represents the assumed-average Fe:Mn ratio of phytoplankton (2.67)[1].

Supplementary Figures

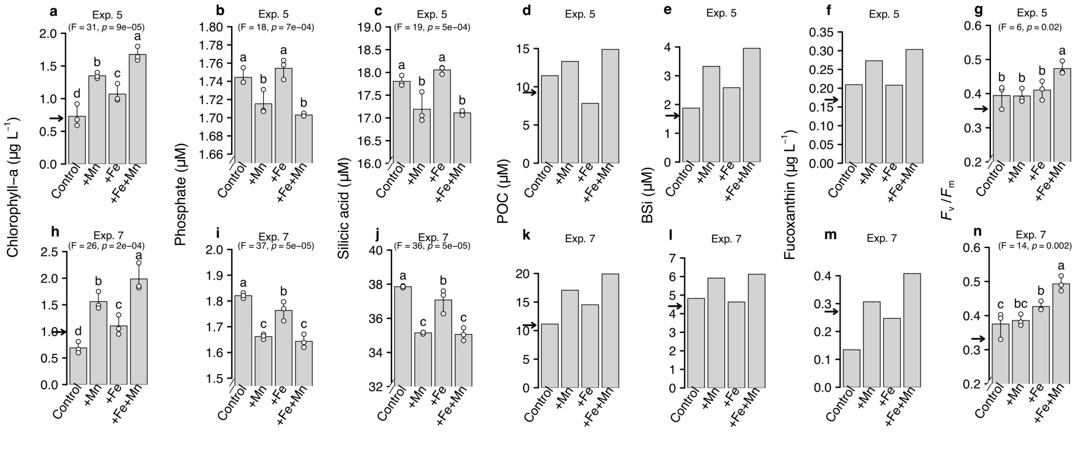

Supplementary Figure 1. Changes in biochemical and physiological parameters at Mn limited Experiments 5 and 7. a–g, Experiment 5; **h–n**, Experiment 7. Where available in triplicate biological replicates, bar heights indicate the mean, white symbols are the individual replicate values (connecting line is the range), and different letters above bars indicate statistically different mean responses (one-way ANOVA, F and *p* values shown, followed by Fisher Least Significant Difference test). Bars with no points or letters have only one replicate (pooled treatments). Arrows indicate average initial values (n=3 for chlorophyll-a and F_v/F_m, n=1 for POC and fucoxanthin concentrations).

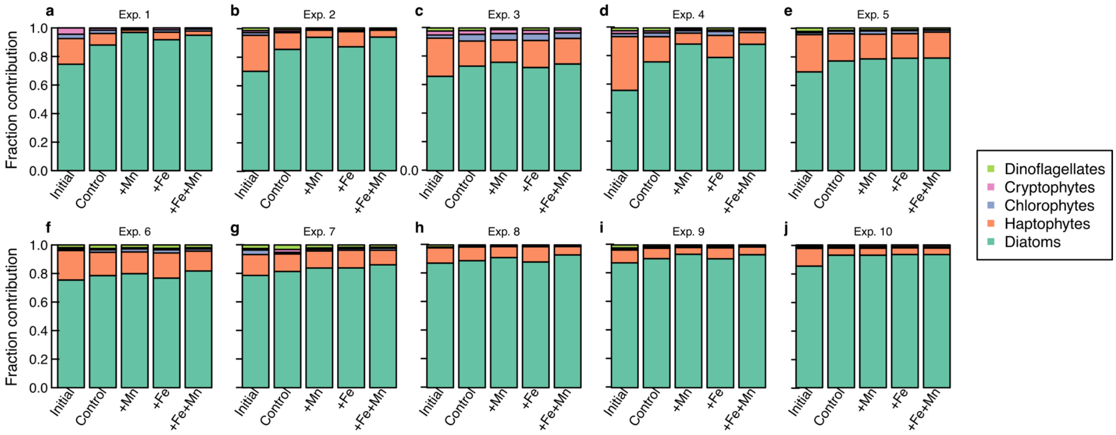

Supplementary Figure 2. Phytoplankton community composition. Predicted contribution of different phytoplankton types to total chlorophyll-a via diagnostic pigment analysis and CHEMTAX.

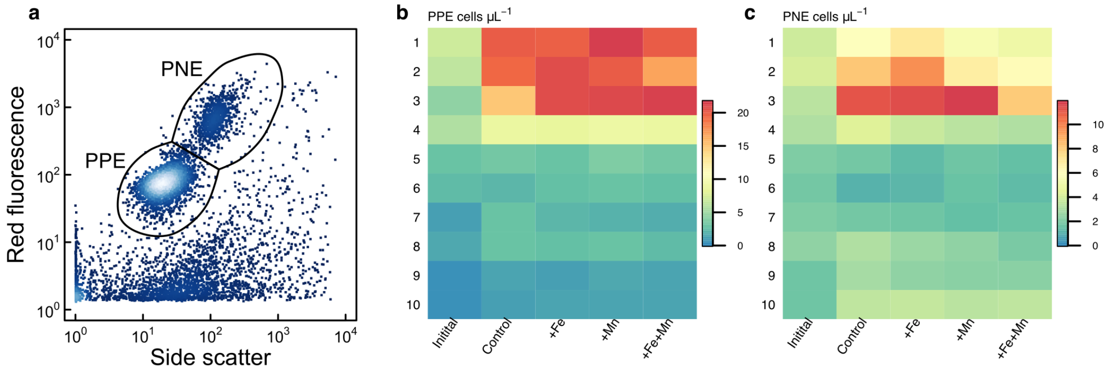

Supplementary Figure 3. Responses of small phytoplankton analyzed by flow cytometry. a, Flow cytometry gating strategy. PPE indicates photosynthetic picoeukaryotes (approximately 0.2–2 µm); PNE indicates photosynthetic nanoeukaryotes (approximately 2–20 µm). Gate positioning was checked and adjusted manually for each sample to account for changes in cellular fluorescence. The units for the axes are arbitrary. **b,** photosynthetic picoeukaryotes. **c,** photosynthetic nanoeukaryotes.

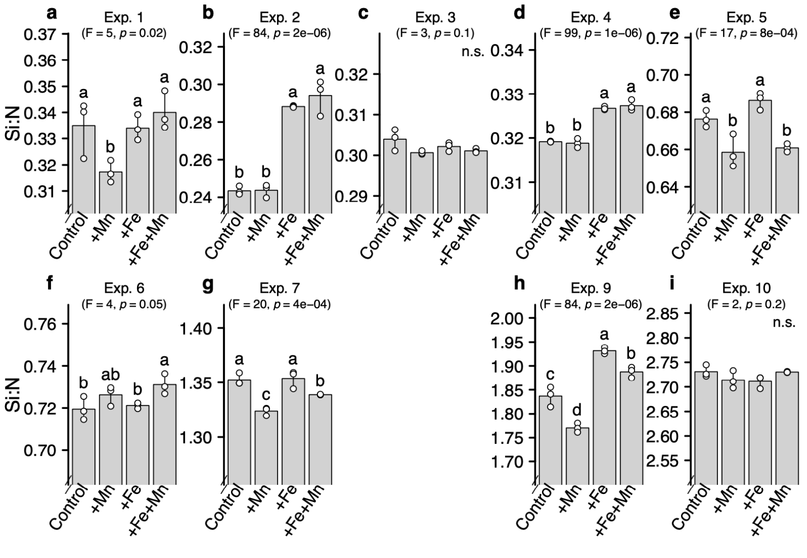

Supplementary Figure 4. Changes in dissolved silicic acid: nitrate concentration ratios (Si:N) in experiments. Bar heights indicate the mean, white symbols are the individual replicate values (connecting line is the range), and different letters above bars indicate statistically different mean responses (n=3 biological replicates; one-way ANOVA, F and *p* values shown, followed by Fisher Least Significant Difference test; n.s. indicates not significant). Results for Experiment 8 are not shown as samples were stored frozen for >1 year prior to analysis (see Methods; they however showed significant silicic acid: nitrate drawdown in +Fe and +Fe+Mn additions relative to controls and +Mn).

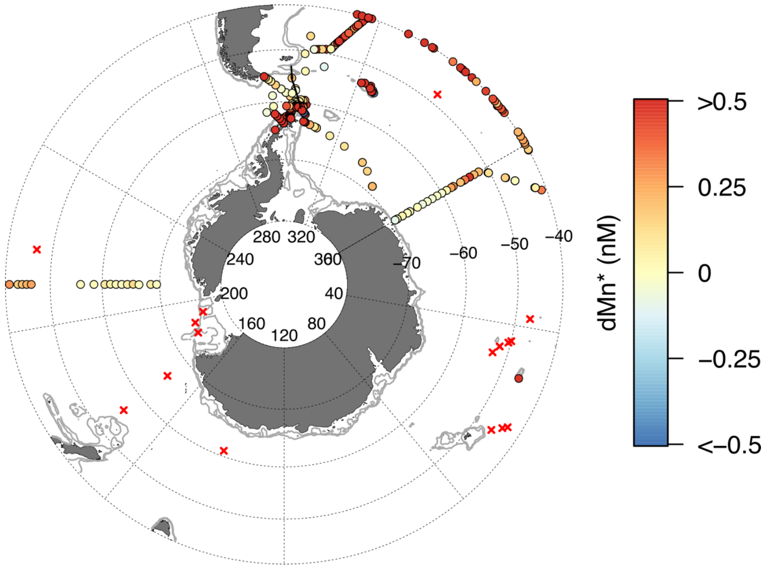

Supplementary Figure 5. Wider extent of Mn deficiency in the surface Southern Ocean. Values are calculated with <30 m depth data from the GEOTRACES Intermediate Data Product[2], CLIVAR[3,4], and Refs 5–11. Yellow points indicate sites approaching Mn-Fe co-deficiency. Red crosses indicate locations where experiments (replicated bottle-scale bioassay experiments or mesoscale enrichment experiments) have found evidence for Fe limitation (from compilation of Ref. 1).

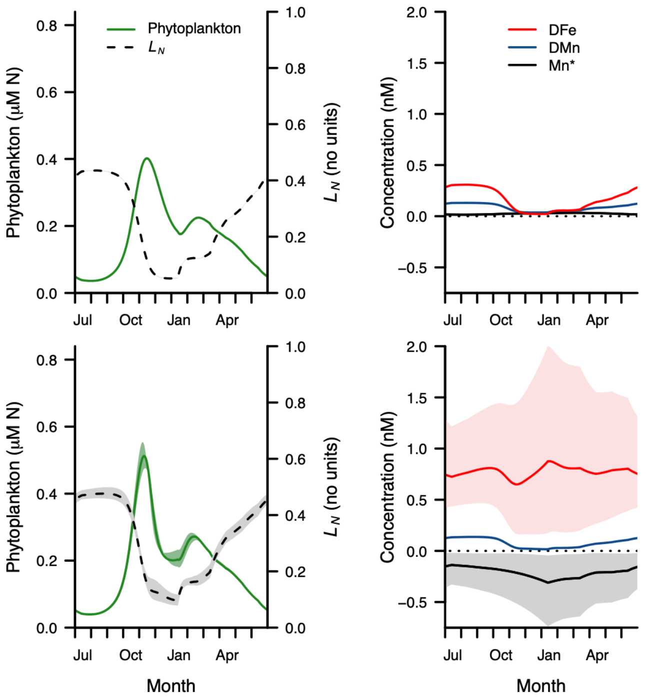

Supplementary Figure 6. Ecosystem model simulation in response to enhanced dust. Details are as for Figure 3d–g and Methods, except (i) all panels are for sites with low open ocean Mn*, isolated deep-water case; and (ii) dust inputs have been included in simulations. Upper panels are for interglacial, low dust conditions (0.014 g m^{-2} yr^{-1})[12]; lower panels are for glacial high dust conditions (3.45 g m^{-2} yr^{-1})[13]. The Fe and Mn content of dust were prescribed (Fe=30,890 p.p.m.; Mn=527 p.p.m.)[14]. Solid lines represent results when solubilities of Fe and Mn are set to the medium values found in the compilation of Ref. 15, whilst shading represents the range when lower and upper quartile values of the compilation are used (see main text and Methods).

Supplementary References

1. Moore, C.M. et al. Processes and patterns of oceanic nutrient limitation. *Nat. Geosci.* **6**, 701-710 (2013).
2. Schlitzer, R., et al. The GEOTRACES intermediate data product 2017. *Chem. Geol.* **493**, 210-223 (2018).
3. van Hulten, M. et al. Manganese in the west Atlantic Ocean in the context of the first global ocean circulation model of manganese. *Biogeosciences* **14**, 1123–1152 (2017).
4. Tagliabue, A., et al. The interplay between regeneration and scavenging fluxes drives ocean iron cycling. *Nat. Commun.* **10**, 4960 (2019).
5. Hatta, M., Measures, C.I., Selph, K.E., Zhou, M. & Hiscock, W.T. Iron fluxes from the shelf regions near the South Shetland Islands in the Drake Passage during the austral-winter 2006. *Deep Sea Res. Pt II* **90**, 89-101 (2013).
6. Measures, C.I., Brown, M.T., Selph, K.E., Apprill, A., Zhou, M., Hatta, M. & Hiscock, W.T. The influence of shelf processes in delivering dissolved iron to the HNLC waters of the Drake Passage, Antarctica. *Deep Sea Res. Pt I* **90**, 77-88 (2013).
7. Martin, J.H., Gordon, R.M. & Fitzwater, S.E. Iron in Antarctic waters. *Nature* **345**, 156-158 (1990).
8. Planquette, H., Statham, P.J., Fones, G.R., Charette, M.A., Moore, C.M., Salter, I., Nedelec, F.H., Taylor, S.L., French, M., Baker, A.R. & Mahowald, N., 2007. Dissolved iron in the vicinity of the Crozet Islands, Southern Ocean. *Deep Sea Res. Pt II* **54**, 1999-2019 (2007).
9. Castrillejo, M., Statham, P.J., Fones, G.R., Planquette, H., Idrus, F. and Roberts, K. Dissolved trace metals (Ni, Zn, Co, Cd, Pb, Al, and Mn) around the Crozet Islands, Southern Ocean. *J. Geophys. Res. Oceans* **118**, 5188-5201 (2013).
10. Browning, T.J. et al. Strong responses of Southern Ocean phytoplankton communities to volcanic ash. *Geophys. Res. Lett.* **41**, 2851-2857 (2014).
11. Schlosser, C., Schmidt, K., Aquilina, A., Homoky, W.B., Castrillejo, M., Mills, R.A., Patey, M.D., Fielding, S., Atkinson, A. and Achterberg, E.P. Mechanisms of dissolved and labile particulate iron supply to shelf waters and phytoplankton blooms off South Georgia, Southern Ocean. *Biogeosciences* **15**, 4973-4993 (2018).
12. Wagener, T., Guieu, C., Losno, R., Bonnet, S. & Mahowald, N. Revisiting atmospheric dust export to the Southern Hemisphere ocean: Biogeochemical implications. *Global Biogeochem. Cy.* **22**, GB2006 (2008).
13. Watson, A.J., Bakker, D.C.E., Ridgwell, A.J., Boyd, P.W. & Law, C.S. Effect of iron supply on Southern Ocean CO 2 uptake and implications for glacial atmospheric CO2. *Nature* **407**, 730-733 (2000).
14. Wedepohl, K.H. The composition of the continental crust. *Geochim. Cosmochim. Acta* **59**, 1217-1232 (1995).
15. Chance, R., Jickells, T.D. & Baker, A.R. Atmospheric trace metal concentrations, solubility and deposition fluxes in remote marine air over the south-east Atlantic. *Mar. Chem.*, **177**, 45-56 (2015).
