# Supplementary material for: Manganese co-limitation of phytoplankton growth and major nutrient drawdown in the Southern Ocean
Source: Nat Commun. 2021 Feb 9;12:884. doi: 10.1038/s41467-021-21122-6 (PMC7873070; doi:10.1038/s41467-021-21122-6)
Supplement: Supplementary file 3 — Reporting Summary [file 41467_2021_21122_MOESM3_ESM.pdf]

nature research

Corresponding author(s): Thomas J. Browning

Last updated by author(s): Dec 22, 2020

Reporting Summary

Nature Research wishes to improve the reproducibility of the work that we publish. This form provides structure for consistency and transparency in reporting. For further information on Nature Research policies, see our Editorial Policies and the Editorial Policy Checklist.

Statistics

For all statistical analyses, confirm that the following items are present in the figure legend, table legend, main text, or Methods section.

n/a	Confirmed	
☐	☒	The exact sample size (n) for each experimental group/condition, given as a discrete number and unit of measurement
☐	☒	A statement on whether measurements were taken from distinct samples or whether the same sample was measured repeatedly
☐	☒	The statistical test(s) used AND whether they are one- or two-sided<br>*Only common tests should be described solely by name; describe more complex techniques in the Methods section.*
☒	☐	A description of all covariates tested
☒	☐	A description of any assumptions or corrections, such as tests of normality and adjustment for multiple comparisons
☐	☒	A full description of the statistical parameters including central tendency (e.g. means) or other basic estimates (e.g. regression coefficient) AND variation (e.g. standard deviation) or associated estimates of uncertainty (e.g. confidence intervals)
☐	☒	For null hypothesis testing, the test statistic (e.g. F, t, r) with confidence intervals, effect sizes, degrees of freedom and P value noted<br>*Give P values as exact values whenever suitable.*
☒	☐	For Bayesian analysis, information on the choice of priors and Markov chain Monte Carlo settings
☒	☐	For hierarchical and complex designs, identification of the appropriate level for tests and full reporting of outcomes
☒	☐	Estimates of effect sizes (e.g. Cohen's d, Pearson's r), indicating how they were calculated

Our web collection on statistics for biologists contains articles on many of the points above.

Software and code

Policy information about availability of computer code

Data collection	Flow cytometry: CellQuest software version 3.3 (Becton Dickenson).<br>High performance liquid chromatography: Chromeleon version 7.0 (Thermo Fisher Scientific).<br>Fast repetition rate florometry: FastPro8 (Chelsea Technologies Group).<br>Particulate organic nitrogen/carbon: Callidus version 5.1 (Eurovector)<br>ICP-MS: ELEMENT 2/XR software version 3.1 (Thermo Scientific)
Data analysis	Statistics and other calculations were conducted using R version 3.2.2.

For manuscripts utilizing custom algorithms or software that are central to the research but not yet described in published literature, software must be made available to editors and reviewers. We strongly encourage code deposition in a community repository (e.g. GitHub). See the Nature Research guidelines for submitting code & software for further information.

Data

Policy information about availability of data

All manuscripts must include a data availability statement. This statement should provide the following information, where applicable:

- Accession codes, unique identifiers, or web links for publicly available datasets
- A list of figures that have associated raw data
- A description of any restrictions on data availability

Source data are provided with this paper. Experimental data are provided in the Supplementary Table 1 and Supplementary Data 1. Supplementary Data 1 contains the data used to produce Figure 2 and Supplementary Figures 1–4. Additional Southern Ocean trace metal datasets used in this study are available in public repositories: the GEOTRACES Intermediate Data Product 2017 via the British Oceanographic Data Centre (https://www.bodc.ac.uk/geotraces/data/idp2017/) and

the Antarctic Peninsula dataset via the Biological and Chemical Oceanography Data Management Office (https://www.bco-dmo.org/dataset/3800/data; https://www.bco-dmo.org/dataset/3801/data).

Field-specific reporting

Please select the one below that is the best fit for your research. If you are not sure, read the appropriate sections before making your selection.

☐ Life sciences ☐ Behavioural & social sciences ☒ Ecological, evolutionary & environmental sciences

For a reference copy of the document with all sections, see nature.com/documents/nr-reporting-summary-flat.pdf

Ecological, evolutionary & environmental sciences study design

All studies must disclose on these points even when the disclosure is negative.

Study description	Field sampling and experiments were conducted onboard the RRS James Clark Ross in November 2018 (JR18002). Seawater was collected under trace-metal-clean conditions using a towed water sampling device (~2 m depth) and filled in 1 L acid-washed polycarbonate bottles (Nalgene). Triplicate amendments of Fe, Mn, and Fe+Mn were performed and were incubated for 2–5 days (see Supplementary Table 1). Additionally, three bottles were incubated with no amendment (controls) and three were sampled for initial conditions. Nutrient, trace element and phytoplankton community structure samples were collected alongside experimental seawater. Following incubation, bottles were sub-sampled for chlorophyll-a concentrations, flow cytometry cell counts, fast repetition rate fluorometry, macronutrient concentrations, diagnostic pigments, particulate organic carbon, and biogenic silicate.
Research sample	Natural mixed assemblages of microbial communities in surface seawaters encountered on the research cruise. We aimed to achieve good across-Drake Passage experimental coverage, but the exact timing and location of sampling was random.
Sampling strategy	No sample size calculation was performed. Nutrient amendment experiments were conducted with triplicate biological replicates, thus allowing for statistical testing whilst remaining logistically feasible in carrying out the field study. The nutrient amendment experiments were conducted at the highest spatial and temporal resolution possible during the oceanographic research cruise.
Data collection	Samples were collected by T. Browning and E. Mawji on the research cruise. Samples were analyzed by T. Browning, E. Mawji, and several technical staff at GEOMAR Helmholtz Centre for Ocean Research Kiel. Instruments used for data collection were:<br>Chlorophyll-a concentrations: Trilogy fluorometer (Turner Designs)<br>Fast Repetition Rate fluorometry measurements: FASTOcean (Chelsea Technologies Group)<br>Flow cytometry measurements: FACSCalibur flow cytometer (Becton Dickenson)<br>Particulate organic carbon measurements: EA3000 Elemental Analyzer (Eurovector)<br>High Performance Liquid Chromatography measurements: Dionex UltiMate 3000 LC system (Thermo Scientific)<br>Macronutrient measurements: AA3 nutrient autoanalyzer (Seal Analytical)<br>Trace element measurements: Element XR (Thermo Scientific)
Timing and spatial scale	Samples were collected between 3rd - 18th November 2018. Sampling for experiments was conducted in between both the activities of other cruise participants and stormy weather conditions. Experimental samples were collected at night time, dawn, or dusk in order that phytoplankton were dark acclimated. The geographic bounds of sample collection were –60.97 N to -54.71 N, -58.01 E to -54.64 E.
Data exclusions	As detailed in the methods, nutrient data from one experiment (of the 10 conducted) were excluded from the analysis as unlike the other samples these were not analyzed on ship but stored frozen for ~1 year before analysis. This exclusion criteria was not pre-established, but decided upon after comparing with the data collected on-ship.
Reproducibility	Identical experiments were conducted 10 times at different locations with treatments having triplicate replication. This was the maximum reproducibility possible during the fieldwork. All attempts at replication were successful.
Randomization	Incubation bottles for the nutrient amendment experiments were filled at random. Allocation of nutrient treatment to each sample was random.
Blinding	Investigators were not blinded to nutrient treatments in that experimental incubation bottles were labeled with the treatment they received. Most subsequent analytical measurements (flow cytometry cell counts, particulate organic carbon concentrations, macronutrient concentrations, pigment measurements, trace element measurements) were allocated independent sample IDs and the analyst was therefore blinded to sample location and/or experiment number/treatment.

Did the study involve field work? ☒ Yes ☐ No

Field work, collection and transport

Field conditions	Encountered seawater temperatures ranged from -0.6 to 5.4 degrees Celsius. Weather and sea conditions varied on the research cruise from calm to storm conditions. In the latter state no sample collection was permitted.
Location	Samples were collected within the following domain: 58.01W to 54.64W, 60.97S to 54.71S. Samples were all collected from the near-sea surface (~2 m depth).

Access & import/export	Samples were acquired and transported back to the United Kingdom adhering to sampling and import/export policies of the British Antarctic Survey (Cambridge, United Kingdom).
Disturbance	Minimal disturbance was generated by the open ocean fieldwork activities (i.e., the presence of the research ship and towing of the seawater sampling device). All chemicals and seawater exposed to chemicals were transported back to the United Kingdom for disposal.

Reporting for specific materials, systems and methods

We require information from authors about some types of materials, experimental systems and methods used in many studies. Here, indicate whether each material, system or method listed is relevant to your study. If you are not sure if a list item applies to your research, read the appropriate section before selecting a response.

Materials & experimental systems

n/a	Involved in the study
☒	☐ Antibodies
☒	☐ Eukaryotic cell lines
☒	☐ Palaeontology and archaeology
☒	☐ Animals and other organisms
☒	☐ Human research participants
☒	☐ Clinical data
☒	☐ Dual use research of concern

Methods

n/a	Involved in the study
☒	☐ ChIP-seq
☐	☒ Flow cytometry
☒	☐ MRI-based neuroimaging

Flow Cytometry

Plots

Confirm that:

☒ The axis labels state the marker and fluorochrome used (e.g. CD4-FITC).

☒ The axis scales are clearly visible. Include numbers along axes only for bottom left plot of group (a 'group' is an analysis of identical markers).

☒ All plots are contour plots with outliers or pseudocolor plots.

☒ A numerical value for number of cells or percentage (with statistics) is provided.

Methodology

Sample preparation	Seawater was collected under trace metal clean conditions, incubated, and sub-sampled for flow cytometry cell counts. Samples (2 mL) were fixed with neutralized paraformaldehyde at a 1% final concentration (paraformaldehyde: methanol-free 16% 10 mL glass ampules, Alfa Aesar/Thermo Fisher), vortex-mixed, and left in the dark for 10 minutes before being transferred to a –80 °C freezer. Samples were thawed at room temperature before analysis.
Instrument	FACSCalibur flow cytometer (Becton Dickenson, Oxford, United Kingdom).
Software	CellQuest software (Becton Dickenson).
Cell population abundance	Identification and counts of phytoplankton populations only (no cell sorting).
Gating strategy	Plots of orange fluorescence versus red fluorescence were used to identify and enumerate Synechococcus from other photosynthetic picoeukaryotes and nanoeukaryotes, and plots of side scatter versus red fluorescence (with any Synechococcus gated out) were used to enumerate photosynthetic nanoeukaryotes and picoeukaryotes. Gates were checked and adjusted manually for every sample to account for variations in fluorescence per cell. Identification of the division between pico- and nanophytoplankton was aided by subsequent analysis of 2 µm filtrates for some samples.

☒ Tick this box to confirm that a figure exemplifying the gating strategy is provided in the Supplementary Information.
